# Case report: Febrile infection-related epilepsy syndrome in a 14-year-old girl with multiple organ failure and lethal outcome

**DOI:** 10.3389/fnins.2024.1255841

**Published:** 2024-03-05

**Authors:** Lars Ruttkowski, Ines Wallot, Marie Korell, Elke Daur, Peter Seipelt, Andreas Leonhardt, Stefanie Weber, Nadine Mand

**Affiliations:** ^1^Pediatric Intensive Care, Department of Pediatrics, Philipps-University Marburg, Marburg, Germany; ^2^Pediatric Neurology, Department of Pediatrics, Philipps-University Marburg, Marburg, Germany; ^3^Pediatric Nephrology, Department of Pediatrics, Philipps-University Marburg, Marburg, Germany

**Keywords:** FIRES, NORSE, pediatric status epilepticus, multiple organ failure, pediatric neurology

## Abstract

We report a case of an otherwise healthy 14-year-old girl with febrile infection-related epilepsy syndrome (FIRES), multiple organ failure (MOF), and ultimately a lethal outcome. This is a rare case of FIRES with MOF and consecutive death. Only a few cases have been described in the literature. The adolescent girl was initially admitted to our pediatric emergency department with a first episode of generalized tonic–clonic seizures after a short history of fever a week before admission. Seizures progressed rapidly into refractory status epilepticus without any evidence of the underlying cause, and treatment subsequently had to be escalated to thiopental anesthesia. Since the initial diagnostics showed no promising leads, the rare syndrome of FIRES was suspected, representing a catastrophic epileptic encephalopathy linked to a prior benign febrile infection. Methylprednisolone, intravenous immunoglobulins, and a ketogenic diet were initiated. Respiratory, circulatory, kidney, and liver failure developed during treatment, requiring increasing intensive care. Multiple attempts to deescalate antiepileptic treatment resulted in recurrent status epilepticus. A cranial MRI on the 10th day of treatment revealed diffuse brain edema and no cerebral perfusion. The patient was declared dead on the 11th day of treatment. FIRES should be taken into account in previously healthy children with a new onset of difficult-to-treat seizures after a short febrile infection when no other cause is apparent. First-line treatment, besides seizure control, is the early initiation of immunomodulatory therapy and the start of a ketogenic diet. As treatment is difficult and MOF may develop, patients should be transferred to a specialized children’s hospital providing full intensive care.

## 1 Introduction

Febrile infection-related epilepsy syndrome (FIRES) is a rare, fever-induced, refractory epileptic encephalopathy with high mortality in otherwise healthy patients. It is seen as a subcategory of new-onset refractory status epilepticus (NORSE), a clinical presentation of severe refractory status epilepticus (RSE) without an acute or active structural, toxic, or metabolic cause identifiable. In contrast to prolonged febrile status epilepticus, fever usually occurs between 2 weeks and 24 h before the onset of RSE (Gaspard et al., 2018).

In Germany, FIRES has an estimated incidence and prevalence of 1:1.000.000 and 1:100.000, respectively (van Baalen et al., 2012). Very few patients show a complete recovery, and most patients continue to suffer from refractory epilepsy, cognitive impairment, behavioral disorders, or learning disabilities with an overall mortality rate of up to 20% (van Baalen et al., 2010; Kramer et al., 2011; Sculier et al., 2021). The etiology and pathophysiology of this severe disorder remain unclear so far. To this day, no structural or infectious triggers have been identified to be associated with FIRES. A dysregulated activation of the innate immune system with overactive proinflammatory cytokines in the central nervous system is discussed (Clarkson et al., 2019), whereas others hypothesize that mesial temporal damage could lead to refractory epilepsy (Koh et al., 2021). In adults, approximately half of cryptogenic NORSE cases were due to immune encephalitis (Lattanzi et al., 2022). Research for potential underlying genetics has been inconclusive. Known genes (e.g., *SCN1A* or *KCNT1*), which are affected in other epileptic disorders, show no abnormality in FIRES/NORSE patients (Lee, 2020; De Danielle et al., 2023). Patients usually present with a sudden onset of recurrent focal or generalized seizures, which often result in a status of epilepticus. Typically, there is no epilepsy or other chronic disease in the medical history, and family history is not indicative. Different febrile infections with flu-like symptoms before the onset of the RSE have been reported, frequently followed by an afebrile and asymptomatic interval of 1 or 2 days (van Baalen et al., 2017; Sculier et al., 2021) Diagnostics may show mild pleocytosis or elevated protein levels in the cerebral spinal fluid (CSF), status epilepticus (SE) in EEG, and non-specific anomalies in cranial MRI (van Baalen et al., 2017; Sculier et al., 2021; Pavone et al., 2022). Therapy is challenging, as seizures are difficult to control (Kramer et al., 2011). A ketogenic diet (KD), steroids, and other immunomodulating drugs (e.g., intravenous immunoglobulins (IVIGs) and IL-1 inhibitors) are empirically used in the treatment of FIRES (Kessi et al., 2020).

Multiple organ failure (MOF) is a rare complication in FIRES associated with poor outcome and survival. Only a few cases of FIRES-associated MOF and consequent death have been published (Arayakarnkul and Chomtho, 2019; Kessi et al., 2020; Soler Wenglein et al., 2023).

We report a fatal case of a 14-year-old girl with severe RSE after a brief history of fever and headache who developed MOF, ultimately leading to brain edema and death.

## 2 Timeline


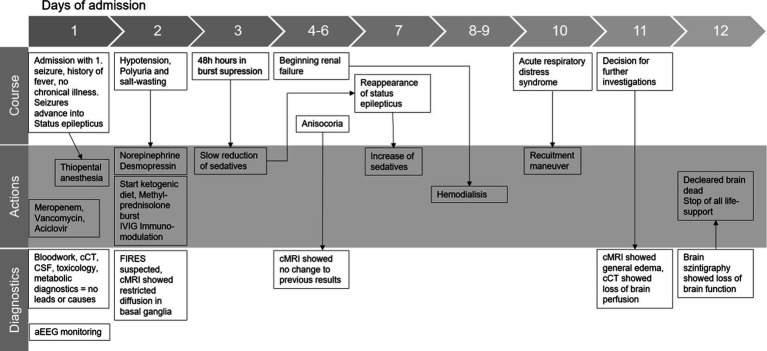


## 3 Case description

A previously healthy 14-year-old girl was admitted to our pediatric emergency department (PED) after a generalized tonic–clonic seizure. In the week before admission, a short episode of fever up to 40°C, headache, and vomiting was reported, from which the patient recovered completely. The caretaker reported a change in character since the day before, with the girl being unusually quiet and keeping mostly to herself. No form of drug abuse or medication intake was reported by the family; vaccination status was complete, the last vaccination being Anti-SARS-CoV-2 by Pfizer 2.5 months before admission. There was no medical or family history of epilepsy or any other chronic illness.

Initial physical examination showed impaired consciousness (GCS 10) and light-responsive pupils. Respiratory and cardiocirculatory status were inconspicuous. Shortly after admission, a second generalized tonic–clonic seizure occurred with gaze and head deviation to the right, fencing posture to the left, incontinence of urine, and smacking of lips. The symptoms subsided after 4 mg (0.05 mg/kg) of intravenous Midazolam. The patient was transferred to the pediatric intensive care unit (PICU). Within the first 12 h, recurrent seizures occurred despite escalating anticonvulsive treatment with repeated single-dose midazolam, levetiracetam (20 mg/kg), phenobarbital (10 mg/kg), and eventually continuous midazolam infusion (4 μg/kg/min). Seizure activity ceased when thiopental was initiated and administered continuously (max. 10 μg/kg/h), with EEG showing a burst suppression pattern. The patient was intubated and on pressure-controlled ventilation. Broadband antibiotics and aciclovir were started. Arterial hypotension was treated with norepinephrine.

The initiated diagnostics were inconclusive: inflammation markers (CrP, procalcitonin, C3/C4 complement) were not elevated, electrolytes, liver enzymes, and extensive lab tests were normal. There was no evidence of toxins, vasculitis, or hyperthyroidism. Cranial CT did not show any pathology. CSF revealed a mild pleocytosis (13 M/L); glucose and lactate were normal, indicating an inflammatory process. Blood culture and CSF culture tested negative for bacteria, and the RT-PCR test on herpes simplex was negative. RT-PCR for common respiratory viruses in an oral swab showed weak evidence of parechovirus. The serological screening revealed no recent infection with adeno-, Epstein–Barr-, measles-, mumps-, rubella virus, varicella zoster, or SARS-CoV-2. Over the course of the first few days, we ruled out other causes of RSE, such as autoimmune, limbic, and herpes-simplex-encephalitis. A cMRI showed restrictions on diffusion in the basal ganglia but no evidence of encephalitis. These findings are shown in other FIRES patients as well (Moreno-Brauer et al., 2023).

As FIRES was suspected, methylprednisolone bursts (1 g/d for 5 days) were administered within 48 h of admission, and a KD (fat-to-carbohydrate ratio of 3:1) was introduced. Thiamin and biotin were added. Although the diet was followed strictly, we were not able to establish a ketogenic metabolic status during the time of treatment. The patients’ general condition temporarily stabilized. After 48 h of seizure-free EEG monitoring, the anticonvulsive thiopental therapy was slowly tapered, but focal seizures reappeared on day 7. Anticonvulsive treatment was therefore re-intensified, and IVIGs (2 g/kg) were added to the therapy. Anakinra was discussed as a therapy option but was held off to evaluate the already started immunomodulating therapies. The patient’s condition deteriorated rapidly within the next few days.

Polyuria and hypernatremia in the context of central diabetes insipidus were treated with crystalloid fluids (up to 4,000 mL per day) and desmopressin. Temperatures up to 39.4°C, which were not responding to antipyretic drugs (metamizole or paracetamol), were treated with external cooling. Rising kidney retention parameters, proteinuria, and reduced renal perfusion were observed from day 4. Initiated treatment with diuretics showed sufficient water diuresis, but still potassium, phosphate, and urea levels increased over time, which ultimately led to the start of hemodialysis on day 8. Hepatic dysfunction occurred with increasingly impaired coagulation, and fresh frozen plasma was administered repetitively from day 8. Catecholamine therapy had to be intensified from day 9 by adding dobutamine and milrinone due to cardiac failure. Additionally, respiratory status declined with clinical and radiological signs of ARDS (min. PaO_2_/FiO_2_ 70) on day 9; ileus and rhabdomyolysis developed. Repetitive laboratory diagnostics were inconclusive. Recurrent cranial MRIs showed reduced diffusion in the basal ganglia. Intracranial bleeding, ischemia, or brain edema were ruled out repetitively, especially after anisocoria appeared for the first time on day 4. On day 10, the cMRI showed diffuse swelling of the entire brain with emphasis on the cerebellum, narrow lateral ventricles, herniation of the cerebellar tonsils, and absent flow in the cerebral vessels. Suspected brain death was confirmed with pathological cranial CT angiography and cerebral perfusion scintigraphy. The patient was declared dead on day 11. Life support measures were terminated after the family’s farewell. An autopsy was not performed due to the wishes of the family.

## 4 Discussion

FIRES is a poorly understood syndrome that is characterized by catastrophic and difficult-to-treat SE, resulting in high morbidity and mortality in children (van Baalen et al., 2010), especially in cryptogenic FIRES cases (Shi et al., 2023). Although the etiology is unknown in most cases, the most frequently found cause of NORSE in adults is autoimmune encephalitis (Lattanzi et al., 2022). Immunomodulatory therapy, e.g., early admission of steroids, IVIG, and use of plasma exchange, is currently recommended as first-line therapy, in addition to a KD and escalating anticonvulsive drugs until no seizure activity is seen in EEG monitoring. A therapy based on the suspected etiology should be administered early. In prolonged cases, an anti-inflammatory treatment including the use of IL-1 inhibitors is to be considered (Wickstrom et al., 2022).

Our patient was admitted with an RSE after a short febrile illness with an asymptomatic interval. FIRES was suspected early, as toxic and metabolic causes for SE, as well as structural diseases or intracranial pathologies, were ruled out. Apart from a positive RT-PCR test on parechovirus in an oral swab, we did not find any infectious causes. Though parechovirus encephalitis has been described in neonates (Sirsi et al., 2023), there was no evidence in our patient as RT-PCR in the CSF was negative. Pleocytosis was the only hint toward an inflammatory process and may occur in FIRES patients. Extensive diagnostics did not reveal signs of autoimmune encephalitis as previously described in other cases (Basso et al., 2022). Though no autopsy was performed, we therefore strongly suspect cryptogenic FIRES in our patient.

Treatment focused on aggressive antiepileptic therapy, establishing a KD, and starting steroids; those therapies were described as most effective at that time (Gaspard et al., 2018; Lin et al., 2020; Baba et al., 2021). However, treatment of seizures in cryptogenic FIRES is described as difficult, with seizure activity lasting several weeks with little or no response to antiepileptic treatment and anesthetics often being required (Rachfalska et al., 2021; Cerovic et al., 2023). Tapering of sedatives and antiepileptic medications resulted in the recurrence of seizure activity in our patient, requiring deep anesthesia with EEG burst suppression. Though prolonged burst suppression reduces cerebral oxygen metabolism in adults (Liu et al., 2023), the long-term outcome seems favorable (Fisch et al., 2023). However, drug-induced burst suppression often resolves seizure activity only temporarily in FIRES (Fox et al., 2017). The KD has been proposed as an efficient and safe treatment for RSE in children (Arya et al., 2018). Although we induced KD within 48 h after admission to our PICU and restricted oral and intravenous glucose intake, a katabolic metabolism was never achieved, with ketone levels rising to a maximum of 0.7 mmoL/L. Previous authors described a similar difficulty in establishing katabolic metabolism in adolescent patients (Nabbout et al., 2023). Based on the possible role of neuroinflammation, steroids and IVIG are used in the treatment of FIRES (Kessi et al., 2020). Methylprednisolone was started early in our patient. Due to a temporary clinical stabilization within the first 3 days after admission, immunomodulatory treatment was not escalated. With RSE reappearing on day 7, IVIGs were started. Further treatment escalation with IL-1 inhibitors, as proposed in current literature (Costagliola et al., 2022; Anima Shrestha et al., 2023; Goh et al., 2023), was discussed, but the patient deteriorated rapidly due to MOF before a decision was made. Earlier application of immunomodulators may improve outcomes, although neurodevelopmental impairment seems to remain unchanged (Wu et al., 2021; Anima Shrestha et al., 2023).

MOF complicated the treatment tremendously. Though FIRES remains a rare and difficult-to-treat syndrome, it is well described in the literature. We did not anticipate MOF, which developed rapidly, with renal, liver, respiratory, and cardiac failure occurring within 3 days after the patient had temporarily stabilized. Though acute kidney impairment is known as an independent factor for outcomes in pediatric patients receiving intensive care (Basu et al., 2015), we suspect an overwhelming inflammatory component of FIRES to be the reason for MOF and, eventually, the death of the patient. However, it is postulated that MOF can be caused by high doses of thiopental, mimicking propofol infusion syndrome, especially in RSE patients (Augoustides et al., 2005). An underlying disease as the cause of the MOF is unlikely but not completely ruled out without genetic analysis and autopsy findings.

To our knowledge, this is one of only a few cases of FIRES with MOF and lethal outcome reported in the international literature. In one other case, a 13.5-year-old boy who also did not respond to a KD or immunomodulatory therapy died of arterial hypotension and MOF (Arayakarnkul and Chomtho, 2019). Though rare, early multimodal immunosuppressive therapy needs to be considered in FIRES. Nonetheless, the course of FIRES as well as its treatment can lead to MOF, which should be anticipated in critically ill patients.

## 5 Conclusion

FIRES is a rare syndrome that should be taken into early consideration when otherwise healthy children with a history of recent fever present with a new onset of seizures and no infectious, toxic, metabolic, or structural cause can be determined. Treatment recommendations emphasize a consequent therapy of SE as well as anti-inflammatory medication and an early beginning of a KD (Wickstrom et al., 2022; Sheikh and Hirsch, 2023; Vinette et al., 2023). Especially immunomodulatory drugs seem to influence outcomes. MOF should be anticipated early on. If MOF occurs in FIRES patients, the prognosis is most likely fatal. The adequate treatment of these complex patients is highly challenging and should therefore be executed in a specialized children’s hospital.

## Data availability statement

The original contributions presented in the study are included in the article/Supplementary material, further inquiries can be directed to the corresponding author.

## Ethics statement

Ethical review and approval was not required for the study on human participants in accordance with the local legislation and institutional requirements. Written informed consent to participate in this study was provided by the participants’ legal guardian/next of kin. The studies were conducted in accordance with the local legislation and institutional requirements.

## Author contributions

LR: Conceptualization, Writing – original draft. IW: Writing – review & editing. MK: Writing – review & editing. ED: Writing – review & editing. PS: Writing – review & editing. AL: Writing – review & editing. SW: Writing – review & editing. NM: Conceptualization, Supervision, Writing – original draft.
